# PainDETECT as a Potential Tool for Personalized Medicine: Predicting Outcome One Year After Knee Arthroplasty

**DOI:** 10.1016/j.mayocpiqo.2025.100649

**Published:** 2025-08-04

**Authors:** Amanda J.W. Wall, Kirsten M. Leyland, Amit Kiran, Nigel K. Arden, Cyrus Cooper, Vishvarani Wanigasekera, M. Kassim Javaid, Andrew J. Price, Irene M.C. Tracey, Anushka Irani

**Affiliations:** a Oxford NIHR Musculoskeletal Biomedical Research Unit, Nuffield Department of Orthopaedics, Rheumatology and Musculoskeletal Sciences, University of Oxford, Botnar Research Centre, Oxford, UK; b Arthritis Research UK Centre for Sport, Exercise and Osteoarthritis, Nuffield Department of Orthopaedics, Rheumatology and Musculoskeletal Sciences, University of Oxford, Botnar Research Centre, Oxford, UK; c Oxford University Centre for Integrative Neuroimaging, Nuffield Department Clinical Neurosciences, University of Oxford, John Radcliffe Hospital, Oxford, UK; d MRC Lifecourse Epidemiology Unit, University of Southampton, UK; e Department of Rheumatology, Mayo Clinic, Jacksonville, FL

## Abstract

**Objective:**

To investigate whether neuropathic-like pain, identified using the PainDETECT questionnaire, predicts postoperative symptoms, using data from 2 independent, prospective cohort studies.

**Patients and Methods:**

Data were collected from patients undergoing primary knee arthroplasty for primary osteoarthritis recruited to the Evaluation of perioperative Pain in Osteoarthritis of the kNEe (EPIONE) Study n=120, from October 1, 2011, to May 30, 2014, and the Clinical Outcomes in Arthroplasty Study (COASt) n=404, from January 1, 2010, to December 31, 2018). The PainDETECT questionnaire score was used to divide patients into nociceptive (<13), unclear (13-18), and neuropathic pain (>18) groups preoperatively using validated cutoffs. As the neuropathic group also captures nociplastic pain, we used neuropathic-like to represent this combination. Surgical outcome was compared between groups using the Oxford Knee Score (OKS) and the presence of moderate to severe pain 12 months after arthroplasty.

**Results:**

Total of 296 (56%) reported nociceptive, 144 (27%) unclear, and 84 (16%) neuropathic-like pain preoperatively. Patients in the neuropathic-like pain group had significantly worse OKS postoperatively, compared with the nociceptive group (34 [12] vs 40 [8], *P*<.05), independent of baseline OKS, age, sex, and body mass index. Moderate to severe pain 12 months after arthroplasty was statistically significantly higher in the unclear (OR 2.19 [95% CI, 1.36-3.53]) and neuropathic-like (OR, 2.83 [95% CI, 1.58-5.09]) pain groups when compared with the nociceptive group.

**Conclusion:**

Patients classified presurgery as having unclear and neuropathic pain by the modified PainDETECT have considerably worse outcomes after surgery. Neuropathic pain categorized by this tool commonly has centralized pain features and is a potential predictor of ongoing postsurgical pain. Knowledge of this may aid informed decision-making with respect to surgical intervention for those with knee osteoarthritis.

Knee osteooarthritis (OA) is characterized by severe knee joint pain, swelling, and stiffness, leading to reduced mobility, function, and quality of life. Total knee arthroplasty is widely regarded as an effective, cost-efficient procedure for the treatment of moderate to severe knee OA. Between 400,000 to 790,000 knee replacements are done each year in the United States,^1^ and around 85,000 total knee replacements are carried out in England and Wales each year.^2^ With an aging population, the demand for costly surgery is predicted to rise substantially.^3^, ^4^, ^5^, ^6^, ^7^ Despite this, 16% to 33% of patients report chronic pain after total knee replacement^8^^,^^9^; it is noted that some patients may be reluctant to report that they have pain after surgery so the actual prevalence is likely even higher than estimated in research studies.^8^ Robust predictors of postoperative outcome are desperately needed to improve decision-making and also identify strategies to improve outcomes after surgery.

Pain can be considered in terms of the 3 main categories: nociceptive, (associated with tissue damage and injury), nociplastic (associated with altered pain processing, often in the central nervous system), and neuropathic (associated with nerve damage and disease).^10^ There is abundant evidence to support the presence of different pain mechanisms between and within individuals with knee OA.^11^, ^12^, ^13^, ^14^, ^15^, ^16^, ^17^ Identifying the leading pain mechanism driving pain in the clinical setting is particularly challenging and, despite considerable efforts,^18^ there are currently no gold-standard methods for diagnosing the different pain subtypes.

The PainDETECT questionnaire (PD-Q) is a low-cost, simple tool which was originally developed and validated to screen for neuropathic pain in patients with lower back pain.^19^ Since then, it has been applied to many painful conditions and we, and others, have reported that the PD-Q may actually serve as a useful indication of centrally mediated pain mechanisms seen in the more recently identified category of nociplastic pain.^13^^,^^15^^,^^16^^,^^20^, ^21^, ^22^ Emerging studies have suggested that a higher PD-Q score may predict worse outcome after arthroplasty.^15^^,^^23^^,^^24^ Furthermore, neuroimaging studies have reported that patients with OA and a high PD-Q score showed considerably greater involvement of pain modulation areas in the brainstem, such as the periaqueductal gray and the rostral ventromedial medulla (RVM), compared with those with a low PD-Q score.^15^^,^^20^ Thus it is likely that the PD-Q can capture features of nociplastic pain, with changes in central pain processing, in addition to neuropathic pain. With this in mind, we use the term neuropathic-like to describe the group of patients with a PD-Q score high enough to be classed as having a high probability of a neuropathic component, according to the original validated cutoff values, but that in this patient population it is likely to be identifying nociplastic pain features. The aim of this study was to investigate whether the presence of neuropathic-like features of pain in primary knee OA pre-arthroplasty, identified using the PD-Q, predicts worse outcome after arthroplasty compared with nociceptive pain. Data from 2 pre-existing, independent prospective observational studies were used to optimize the generalizability of the findings.

## Patients and Methods

We used data from 2 independent prospective cohort studies of patients with primary knee arthroplasty (from October 01, 2011 to May 31, 2014). The first was the Evaluation of perioperative Pain In Osteoarthritis of the kNEe or EPIONE Study, which offered detailed pain characteristics at baseline and additional patient reported outcome measures at an interim timepoint. Neuroimaging data from a subset of the cohort have been previously published.^15^ The second was from the Clinical Outcomes in Arthroplasty Study or COASt study, which offered increased power for multivariate analysis.^25^, ^26^, ^27^

### EPIONE Study

#### Setting and Patient Recruitment

The EPIONE Study was conducted at the Nuffield Orthopaedic Center, as part of Oxford University Hospitals NHS Trust, a specialist referral center for joint replacement surgery. Patient assessment took place at their routine preoperative assessment clinic appointment within 6 weeks of knee arthroplasty (baseline) and the study took place from October 1, 2011, to May 30, 2014. A subgroup of patients were invited to participate in a neuro-imaging substudy, published elsewhere.^15^ The local ethics committee approved the study (NRES Committee-South Central-Oxford B, 09/H0605/76).

#### Data Collection

The recruitment process and study visits for EPIONE are outlined in Figure. Data collection took place at (baseline), at 2 months postoperatively to coincide with the routine clinical follow-up appointment, and at 12 months postoperatively to capture long-term outcome data. The follow-up assessments were self-completed and conducted by post.

**Figure:**
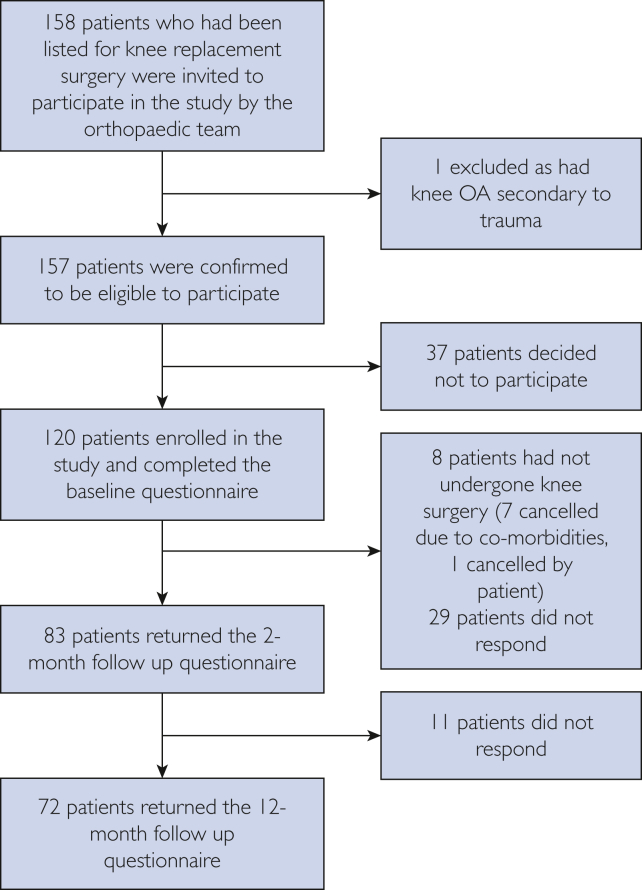
Flow chart of study recruitment and follow-up visits for the EPIONE study. OA, osteoarthritis; QST, quantitative sensory testing.

At baseline demographic characteristic data were collected. Further clinical data were collected from the hospital’s electronic patient clinical record system including medications before surgery. Weight-bearing anterio-posterior films were taken as part of routine clinical care and scored using the Kellgren and Lawrence global score^28^^,^^29^ by a single observer (K.M.L.) who was blinded to patient identity and symptoms. Radiographic severity was dichotomized using a Kellgren and Lawrence score of grade 3 or higher to identify subjects who at least had definite osteophytes and joint space narrowing.^29^

#### Pain Assessment

The presence of neuropathic-like qualities of pain was assessed using the modified PainDETECT questionnaire (mPD-Q), a form of the screening tool, which has been specifically modified for use in knee OA, whereby patients are directed to report the symptoms they are experiencing in their knee.^11^^,^^30^, ^31^, ^32^ The mPD-Q score was used to divide patients, according to established cutoff values, into those with nociceptive (<13), unclear (13-18), and neuropathicpain (>18).^11^^,^^13^^,^^14^^,^^19^ The group classed as being likely to have neuropathic pain were referred to as the neuropathic-like group in view of the evidence suggesting that these features are likely to represent nociplastic pain, as discussed above.

Participants also completed the following questionnaires: the short form of the McGill pain questionnaire,^33^^,^^34^ the hospital anxiety and depression scale, ^35^ the state/trait anxiety inventory,^36^ the pain catastrophizing scale,^37^ the Tampa scale for Kinesiophobia,^38^ the revised life orientation test,^39^ and the Pittsburgh sleep quality index.^40^

### COASt Study

#### Setting and Patient Recruitment

Eligible patients who were placed on the waiting list for knee arthroplasty were recruited across 2 hospitals for this study which was conducted between January 01, 2010 and December 31, 2018: Southampton University Hospital NHS Foundation Trust and Nuffield Orthopaedic Center as part of Oxford University Hospitals NHS Trust. Patient demographic characteristics and clinical data were collected at the preoperative outpatient visit before surgery and annual postoperative follow-up by post for 5 years thereafter. In line with the EPIONE study, the cohort from the COASt study was restricted to the patients who were listed for knee arthroplasty and had 12 month follow-up data available. The COASt study obtained ethical approval from Oxford Research Ethics Committee (REC Ref: 10/H0604/91).

#### Data Collection

Recruitment and data collection in the COASt study has been previously reported.^26^^,^^27^^,^^41^ For this study, baseline data for age, sex, BMI, employment status, side predominantly affected, duration of symptoms, hospital anxiety and depression scale, Euroqol 5 Dimensions, and the type of surgery planned were extracted.

#### Pain Assessment

Pain assessment and the Oxford Knee Score (OKS) were reported before surgery and 12 months after surgery using the mPD-Q.

### Primary Outcome (Both Studies)

We used the OKS, a 12-item composite score developed to measure patient reported outcome after knee arthroplasty, which measures 3 symptom domains: pain, stiffness, and functional disability, in relation to the knee.^42^ It gives a summary score ranging from 0 (worst possible score, most severe symptoms) to 48 (best possible score, least symptoms).^43^ Patients completed the OKS at baseline and at 12 months postoperatively with additional data at 2 months postoperatively in EPIONE only. The primary outcome for this study was the difference in OKS between the neuropathic, unclear, and nociceptive pain groups at 12 months postoperatively in both the EPIONE Study and the COASt study. These analyses were initially investigated separately for each individual study and then combined in a pooled analysis.

### Secondary Outcomes (Both Studies)

We created a binary outcome (no, yes) for patients achieving a 7 point improvement in OKS from baseline, which is the minimally clinically important change in OKS at the patient level.^44^

We also measured pain independently from the OKS by defining moderate to severe long-term pain after surgery as an average pain severity score of 3 or more 12 months after surgery. This was measured using the numeric pain rating average pain severity score, captured using the mPD-Q.^45^ This rating does not contribute to the overall score used to determine whether neuropathic-like features of pain are likely to be present or not.

### Statistical Analyses

All analyses were conducted separately for EPIONE and the COASt study initially and then combined in a pooled analysis. For each cohort, descriptive statistics were used to describe the demographic characterisitcs and clinical baseline data in each of the 3 pain groups. These comprised mean (standard deviation), median (interquartile range), and proportions for normal continuous, non-normal continuous, and categorical data, respectively.

The difference in OKS between those with nociceptive, unclear, and neuropathic pain was reported at 12 months postoperatively in the EPIONE Study and the COASt study separately and then combined, using analysis of covariance to assess for any statistically significant differences adjusting for baseline OKS. A further multivariable model was fitted including age, sex, and BMI. Regression diagnostics checking for normality of residuals, collinearity, homoscedasticity, and linearity were satisfied. The analyses were restricted to patients who had completed assessments at both time points.

For the secondary objectives, we reported the number (percentage) of patients who achieved the minimally clinically important change in OKS of 7 points, (OKS at 12 months minus OKS at baseline). This was reported for each of the 3 pain groups, for each study separately and then combined; differences between the 3 pain subgroups were assessed using χ^2^ test.

We used multivariable logistic regression to determine the association between moderate to severe long-term pain after surgery and pain grouping. The model build adjusted for baseline severity alone (measured using the numeric pain rating average pain severity score, captured using the mPD-Q at baseline), and then for age, sex, and BMI.

A *P*-value of *P*<.05 was considered to be statistically significant throughout. Complete case analyses were conducted based on the assumption that data were missing at random. All analyses were conducted using Stata SE v12.0 (StatCorp).

## Results

### EPIONE Study

Among the 120 patients recruited to the EPIONE study, 25 (21%) had an mPD-Q score characterized as predominantly neuropathic-like pain, 32 (27%) unclear pain, and 63 (53%) nociceptive pain before surgery. Baseline demographic characteristics and clinical features were broadly similar across the 3 pain groups (Table 1). The most striking differences were seen for the McGill pain questionnaire score, additional pain locations, the Kellgren and Lawrence score, and almost all psychological scales, including anxiety, depression, catastrophizing, and sleep quality.

**Table 1:** Preoperative Patient Characteristics for EPIONE AND COASt by Nociceptive, Unclear, and Neuropathic Pain Groups^a^^,^^b^

Preoperative characteristics	EPIONE (n=120)			COASt (n=404)		
	Nociceptive pain	Unclear pain	Neuropathic pain	Nociceptive pain	Unclear pain	Neuropathic pain
	n=63 (53%)	n=32 (27%)	n=25 (21%)	n=233 (58%)	n=112 (28%)	n=59 (15%)
Demographic features						
Age (y), mean ± SD	72 ± 8	68 ± 8	70 ± 10	70 ± 9	67 ± 9	66 ± 9
BMI (kg/m^2)^, mean ± SD	29.5 ± 5.1	30.2 ± 5.2	31.8 ± 4.9	29.6 ± 4.9	31.2 ± 5.6	31.5 ± 5.5
Female, n (%)	27 (43)	20 (63)	14 (56)	116 (50)	62 (55)	37 (63)
Employed, n (%)	14 (22)	12 (38)	3 (13)	68 (29)	41 (37)	18 (31)
Married or living with partner, n (%)	47 (75)	18 (58)	15 (60)	-	-	-
Clinical features						
Duration of pain (mo), median (IQR)	36 (15-90)	60 (24-120)	48 (36-120)	-	-	-
Use of pain-modifying medication, n (%)	36 (57)	22 (69)	17 (68)	-	-	-
SF-MPQ total score (range 0-45), mean ± SD	16.3 ± 9.5	23.0 ± 9.4	24.5 ± 7.7	-	-	-
Right knee affected, n (%)	35 (55)	18 (56)	10 (43)	129 (55)	56 (50)	29 (49)
Number of additional painful body areas for at least 3 months: n (%)						
0	19 (30)	15 (47)	7 (28)			
1-2	27 (42)	5 (15)	7 (28)	-	-	-
≥3	17 (27)	12 (37)	11 (44)	-	-	-
Kellgren and Lawrence grade, n (%)						
0-2	4 (7.1)	6 (20.7)	4 (17.4)	-	-	-
3-4	52 (92.9)	23 (79.3)	19 (82.6)	-	-	-
Psychological characteristics						
HAD anxiety (range 0-21), mean (SD)^c^	6.44 (4.2)	7.2 (5.0)	9.3 (4.0)	4.4 (3.9)	6.4 (3.9)	6.0 (2.9)
HAD depression (range 0-21), mean ± SD^c^	6.3 ± 3.3	7.3 ± 4.8	8.6 ± 4.2	4.2 ± 3.0	5.3 ± 3.6	5.0 ± 3.1
STAI State anxiety range (20-80), mean ± SD	34.0 ± 12.5	39.7 ± 14.2	41.0 ± 14.7	-	-	-
STAI Trait anxiety (range 20-80), mean ± SD	33.4 ± 10.7	37.3 ± 12.9	43.2 ± 15.9	-	-	-
Pain catastrophizing score (range 0-52), median (IQR)	11 (6-17)	19 (10-28)	21 (10-36)	-	-	-
Life orientation Test-R (range 0-24), mean ± SD	16.8 ± 4.3	15.4 ± 5.5	12.5 ± 5.9	-	-	-
Pittsburgh sleep quality index (range 0-21)^d^, mean ± SD	8.6 ± 3.3	10.0 ± 3.9	10.8 ± 4.0	-	-	-
Tampa scale of kinesophobia (range 17-68), mean ± SD	38.3 ± 9.8	39.7 ± 7.6	42.4 ± 4.9	-	-	-

a Abbreviation: IQR, interquartile range.

b The PainDETECT questionnaire was used to divide patients into those with nociceptive (<13), unclear (13-18) and neuropathic pain (>18).

c HAD data were only available for 171 of COASt participants.

d Measures of Pittsburgh Sleep Quality Index were only available for 49, 23, and 20 EPIONE participants in the nociceptive, unclear and neuropathic pain groups, respectively.

Among all participants, 83 (69%) and 72 (60%) had OKS at 2 months and 12 months, respectively. Although there was a trend toward the neuropathic-like pain group having a lower OKS 12 months postoperatively (Table 2), there was no statistically significant association between preoperative nociceptive, unclear, and neuropathic-like pain grouping and OKS 12 months postoperatively (Table 3).

**Table 2:** Summary of OKS and Pain Outcomes for EPIONE and COASt Before and After Surgery^a^

EPIONE	Nociceptive pain (n=63)^b^	Unclear pain (n=32)^b^	Neuropathic pain (n=25)^b^
OKS preop, (mean ± SD) p25,p50,p75	(21 ± 7) 15,20,26	(19 ± 8) 14,19,25	(13 ± 6) 10,13,15
OKS 2-mo postop, (mean ± SD) p25,p50,p75	(37 ± 8) 30,40,43	(34 ± 7) 29,35,40	(30 ± 11) 18,32,41
OKS 12-mo postop, (mean ± SD) p25,p50,p75	(40 ± 8) 38,43,46	(40 ± 7) 35,44,45	(35 ± 12) 32,39,43
OKS MCIC of 7 points (preop to 12-mo), n (%)	37/42 (88)	15/16 (94)	12/14 (85)
Long-term pain after arthroplasty, n (%)	6/42 (14)	6/16 (38)	5/14 (36)

COASt	Nociceptive pain (n=233)^c^	Unclear pain (n=112)^c^	Neuropathic pain (n=59)^c^
OKS preop, (mean ± SD) p25,p50,p75	(22 ± 8) 17,23,27	(19 ± 7)15,19,24	(16 ± 5)13, 15,19
OKS 12-mo postop, (mean ± SD) p25,p50,p75	(40 ± 8) 35.0, 42, 46	(36 ± 10) 30, 39, 44	(33 ± 12) 25, 37, 43
OKS MCIC of 7 points (preop to 12-mo), n (%)	199/219 (91)	89/107 (83)	46/58 (79)
Long-term pain after arthroplasty, n (%)	53/219 (24)	44/107 (41)	29/58 (50)

a Abbreviations: MCIC, minimally clinically important change; OKS, Oxford Knee Score.

b Number at baseline; at 2-month visit there were 46, 23, and 14 patients in the nociceptive, unclear and neuropathic groups respectively. At 12-months there were 42, 16, and 14 patients in the nociceptive, unclear and neuropathic groups respectively.

c Number at baseline; at 12-month visit there were 219, 107, and 58 patients in the nociceptive, unclear and neuropathic groups respectively.

**Table 3:** ANCOVA Models to Investigate Differences in 12-Month OKS Between Pain Groups, Determined Preoperatively^a^

Pain group comparison	EPIONE Study (n=72)				COASt Study (n=384)				Pooled analysis (n=456)			
	Univariate model		Multivariate model		Univariate model		Multivariate model		Univariate model		Multivariate model	
	Effect size (95% CI)	SE	Effect size (95% CI)	SE	Effect size (95% CI)	SE	Effect size (95% CI)	SE	Effect size (95% CI)	SE	Effect size (95% CI)	SE
Unclear vs Nociceptive	−0.45 (−6.81 to 5.92)	2.59	−0.06 (−6.75 to 6.62)	2.72	−2.12 (−4.63 to 0.38)	0.12	−1.85 (−4.45 to 0.64)	1.04	−2.00 (−4.31 to 0.31)	0.96	−1.72 (−4.04 to 0.58)	0.96
Neuropathic vs Nociceptive	−2.56 (−9.79 to 4.67)	2.95	−2.15 (−9.75 to 5.45)	3.10	−3.87 (−7.10 to −0.63)^b^	1.35	−3.73 (−7.00 to −0.47)^b^	1.35	−3.53 (−6.47 to −0.59)^b^	1.22	−3.25 (−6.20 to −0.30)^b^	1.23
Neuropathic vs Unclear	−2.21 (−10.52 to 6.29)	3.42	−2.09 (−10.79 to 6.62)	3.54	−1.74 (−5.17 to 1.68)	1.43	−1.87 (−5.26 to 1.51)	1.41	−1.53 (−4.67 to 1.61)	1.31	−1.53 (−4.64 to 1.58)	1.29

a Univariate model is adjusted for baseline Oxford Knee Score, multivariate model is further adjusted for age, sex and BMI.

b *P*<.05.

There was no statistically significant difference in the number (percentage) of patients who achieved OKS MCIC across the pain groups (χ^2^ test, *P*=.76). Among 72 patients with follow-up data at 12 months, 17 (24%) had moderate to severe long-term pain after surgery. Of those with nociceptive pain before surgery, 6 of 42 (14%) reported moderate to severe long-term pain after surgery compared with 6 of 16 (38%) in the unclear group, and 5 of 14 (36%) in the neuropathic-like pain group (χ^2^ test, *P*=.08). Compared with the nociceptive group, patients in the unclear and neuropathic-like pain groups had higher odds of moderate to severe long-term pain after knee arthroplasty at 12-months postoperatively (Table 4). These estimates were of borderline significance with the majority of the confidence interval above the reference point of 1.

**Table 4:** Logistic Regression Model to Identify the Association Between Pain Group at Baseline and Moderate to Severe Long-Term Pain After Arthroplasty at 12-Month Follow-Up Assessment

Study	Pain group	Univariate model^a^	+Adjusted for age, sex, and BMI
		OR (95% CI)	OR (95% CI)
EPIONE (n=72)	Nociceptive	1	1
	Unclear	3.40 (0.89-13.01)	3.44 (0.86-13.77)
	Neuropathic	2.65 (0.59-11.96)	3.26 (0.80-13.27)
COASt (n=384)	Nociceptive	1	1
	Unclear	2.15 (1.30-3.55)	2.04 (1.22-3.40)
	Neuropathic	3.15 (1.67-5.93)	3.06 (1.60-5.81)
Pooled analysis (n=456)	Nociceptive	1	1
	Unclear	2.23 (1.43-3.65)	2.19 (1.36-3.53)
	Neuropathic	2.95 (1.66-5.26)	2.83 (1.58-5.09)

a Univariate model: adjusted for baseline pain severity score only.

### COASt Study

In the COASt study, there were 404 patients with preoperative data available. Of these 59 (15%) had neuropathic-like pain, 112 (28%) unclear pain, and 233 (58%) nociceptive pain at baseline. The preoperative characteristics for the participants were similar across the pain groups except for age, BMI, and depression (Table 2).

Among all participants, 384 (95%) had OKS at 12 months. There was a statistically significant difference in OKS between the neuropathic-like and nociceptive groups whereby the neuropathic-like group had a worse score 12 months postoperatively (Table 3). Similarly, a lower proportion of the neuropathic-like group achieved OKS MCIC at 12 months after surgery (χ^2^ test, *P*=.03).

Both the unclear and neuropathic-like pain groups had statistically significantly higher odds of moderate to severe long-term pain at 12 months, compared with the nociceptive group, (Table 4). The association remained significant when adjusted for baseline severity, age, sex, and BMI.

### Pooled Analyses

A total of 524 patients with preoperative data available , and of these 456 (87%) had OKS at 12 months. There was a statistically significant difference in OKS between the neuropathic-like and nociceptive groups with the neuropathic-like group having a worse OKS 12 months postoperatively (Table 3). The number (percentage) of patients who achieved OKS MCIC across the pain groups was also significantly different: 236 (90%) of the nociceptive-like group, 104 (85%) of the unclear group, and 58(81%) of the neuropathic-like group, (χ^2^ test, *P*=.04).

Patients in both the unclear and neuropathic-like pain group, compared with the nociceptive group, had statistically significantly higher odds of moderate to severe long-term pain after knee arthroplasty at 12 months postoperatively (Table 4). This association remained significant when adjusted for baseline pain severity, age, sex, and BMI.

## Discussion

This is one of a growing number of studies emphasizing the importance of assessing pain phenotype in knee OA patients before surgery, to predict outcome and optimize treatment. The key findings of this study are that, even in a large sample size, patients with a high mPD-Q score (indicating neuropathic-like pain) had significantly worse OKS 12 months after surgery when compared with those with a low mPD-Q score (nociceptive pain), in addition to a higher odds of moderate to severe long-term pain after knee arthroplasty, independent of other predictors for poor outcome after surgery. The replication of previous finding^23^^,^^24^ in this larger dataset further extends the generalizability of.results and together builds a stronger case for the role of the PD-Q as a robust clinical tool to help predict outcome after knee arthroplasty.

Other measures of neuropathic features have also been shown to be a useful indicator in the postoperative period. For example, using the Douleur Neuropathique 4 and brief pain inventory questionnaires, Lavand'homme et al^46^ reported that the presence of neuropathic features in the early postoperative period was a predictor of persistent postoperative pain 3 months after knee arthroplasty. Bertram et al,^47^ using the Douleur Neuropathique 4 and PD-Q, found that although mean neuropathic pain scores improved between 3 and 15 months, up to half continued to report painful neuropathic symptoms at 15 months after knee arthroplasty.

Similarly, quantitative sensory testing (QST) is an established set of tools used to quantify the somatosensory function in the peripheral and central nervous system. Studies have reported that pretreatment QST can predict pain outcomes after standard OA treatments^24^^,^^48^; however, this is not a consistent finding across all studies.^49^^,^^50^ A further limitation of using QST in routine clinical practice is that it is time-consuming, requires expensive equipment, and also depends on specialized training and expertise to conduct the testing.

The results of this study are further supported by findings by groups who have investigated the effect of co-existing fibromyalgia on outcome after arthroplasty. Fibromyalgia is a typical example of nociplastic pain associated with augmented central nervous system pain-processing^51^ and it has been shown that patients with fibromyalgia have increased risk of complications, and inferior outcomes after knee arthroplasty.^52^^,^^53^ Furthermore, a higher fibromyalgia survey score, indicative of centrally augmented pain, in those undergoing hip and knee arthroplasty has also been shown to predict poorer arthroplasty outcomes and increased postoperative opioid consumption, even among individuals whose score falls well below the threshold for the categorical diagnosis of fibromyalgia.^54^

It has been proposed that centrally augmented pain in a subgroup of patients with OA is responsible for this differential response to surgery. Consistent with this hypothesis, centrally mediated pain sensitization has repeatedly been reported in patients with neuropathic-like pain.^15^^,^^20^ It is understood that central sensitization seen in conjunction with neuropathic-like features of pain in OA is partly mediated by changes in the brainstem RVM and periaqueductal gray components of the descending pain modulatory system.^55^^,^^56^ The RVM pain facilitation cells express the mu opioid receptor, whereas pain inhibitory cells express the kappa opioid receptor.^57^ Imbalance in the RVM to favor net descending facilitation may be a mechanism that contributes to central sensitization in patients with OA, linking activity in the descending pain modulation system to neuropathic-like features. Epigenetic modifications can, of course, also be involved in the development and maintenance of chronic neuropathic^58^ or after surgical pain.^59^

Taken together, and coupled with the mechanistic evidence of central sensitization in patients who score highly on the PD-Q,^15^^,^^20^ these findings collectively indicate an opportunity to optimize outcomes in this subgroup of patients through targeted treatments. Such therapeutic options could include non-pharmacological approaches (such as education, exercise therapy, and cognitive behavioral therapy),^60^, ^61^, ^62^, ^63^ which represent evidence-based strategies for the management of nociplastic pain,^51^^,^^64^ or pharmacological treatments such as duloxetine and amitriptyline.^65^ Furthermore, the PD-Q has been used to stratify patients with end-stage OA knee to effectively treat them with the selective serotonin and norepinephrine reuptake inhibitor duloxetine preoperateively^66^; these authors hypothesize that joint replacement surgery could be postponed in a subset of patients, or could even no longer be needed, reducing health care burden and costs. There are numerous systematic reviews highlighting the benefits of using duloxetine in the perioperative and postoperative period to improve outcomes after total knee arthroplasty,^67^^,^^68^ If a subgroup of patients with a high PD-Q score can be identified and treated preoperatively, reducing central sensitivity, then in theory this should improve postsurgical pain; however, results are currently mixed and further data are awaited.^66^^,^^69^

The main strengths of this study is the use of prospective, longitudinal data to investigate the relationship between preoperative neuropathic-like pain and short-term and long-term outcome in 2, large independent cohorts. The use of repeated measures over time, embedded within the existing hospital care pathway, is also a strength and indicates the feasibility of using the PD-Q in a clinical setting. The main limitation is the fact that both datasets are from a similar geographical region and health care setting. There is a theoretical possibility that a floor and ceiling effect associated with the OKS could bias the results toward the null hypothesis; however, data from the NHS PROMs database suggest that may not be as big a problem as previously suspected. A further limitation is the lack of data on other commonly used methods to identify nociplastic pain, such as the Central Sensitization Inventory or QST. However, we note that there is no current gold-standard method for identifying nociplastic pain in the clinical setting: the current proposed diagnostic criteria are based on expert opinion and require validation.^18^ Finally, additional data providing insights about longer-term outcomes, at or beyond 2 years after surgery, would have provided further confidence in the results of this study.

## Conclusion

This study has shown that the subgroups of patients with unclear or neuropathic-like pain, as identified using the mPD-Q, have significantly worse outcome at 12 months postoperatively compared with those with nociceptive pain. These patients may benefit from increased awareness of their projected outcome to aid informed decision-making with respect to surgical intervention. Future studies investigating the potential role of targeted treatment to optimize outcome in this patient subgroup are still needed.

## Potential Competing Interests

The authors report no competing interests.

## Ethics Statement

Both studies included in this analysis received approval by the ethics committee and written consent was obtained from each participant (NRES Committee-South Central-Oxford B, 09/H0605/76 and REC Ref: 10/H0604/91).
